# A Healthy Balance of Plasma Cholesterol by a Novel Annurca Apple-Based Nutraceutical Formulation: Results of a Randomized Trial

**DOI:** 10.1089/jmf.2016.0152

**Published:** 2017-03-01

**Authors:** Gian Carlo Tenore, Domenico Caruso, Giuseppe Buonomo, Maria D'Avino, Pietro Campiglia, Luciana Marinelli, Ettore Novellino

**Affiliations:** ^1^ Department of Pharmacy, University of Naples Federico II, Naples, Italy.; ^2^ Department of Internal Medicine, Hospital Cardarelli, Naples, Italy.; ^3^ Coop. Samnium Medica, Benevento, Italy.; ^4^ Department of Pharmaceutical and Biomedical Sciences, University of Salerno, Salerno, Italy.

**Keywords:** *Annurca apple*, *nutraceutical*, *plasma cholesterol*, *polyphenols*, *randomized controlled clinical trial*

## Abstract

Cardiovascular diseases are nowadays preferential targets of preventive medicine through a straightforward therapy on lipid profile. However, statins, the first-line lipid-lowering drug therapy, specifically act on low-density lipoprotein cholesterol (LDL-C), having a modest effect on plasma high-density lipoprotein cholesterol (HDL-C) concentrations. Today, a number of novel HDL-targeted therapies are emerging, along with unexpected side effects. Thus, novel and possibly safe substances, able to correct impaired lipid profile in humans, are still in great demand. Herein, based on encouraging clinical data, we formulated a nutraceutical product (AppleMetS^®^, AMS), based on a polyphenolic extract from Annurca apple, and demonstrated that two capsules a day of AMS, after one month, have a LDL-C lowering outcome equivalent to 40 mg of simvastatin or 10 mg of atorvastatin. Nevertheless, different from statin-based therapy, AMS exerted a notable effect on HDL (+49.2%). Based on the trial results, we can assert that AMS formulation could effectively integrate the current therapeutic arsenal to correct impaired lipid profile in humans. Specifically, AMS may be considered a complementary and/or alternative safe substance suitable for the treatment of mildly hypercholesterolemic subjects who do not present occurrence of atheromatous plaques yet.

## Introduction

Atherogenic dyslipidemia, a pathological imbalance of circulating lipoproteins such as low-density lipoproteins (LDLs), very LDLs, or, over others, high-density lipoproteins (HDLs), is a major risk factor for the development of cardiovascular diseases (CDs).^1^ Such imbalance causes an endothelial dysfunction that in turn results in low-grade, chronic blood vessel inflammation, which triggers excessive production of prooxidant factors, a key event in the pathophysiology of atherosclerosis. Later, endothelial injuries involve massive penetration and intimal retention of LDLs, thus making these lipoproteins a major substrate for oxidation by arterial wall cells.^2,3^ Oxidized LDLs are known to be responsible for the atherogenesis acceleration.^4^

Conversely, HDLs stimulate cholesterol transport from the arterial wall to the liver for excretion,^5^ and, having antioxidative, anti-inflammatory, antiapoptotic, antithrombotic, and vasodilator properties, have been regarded as antiatherogenic lipoproteins.^6,7^ Several autonomous studies revealed that low HDL-cholesterol (HDL-C) levels constitute an independent and predictive risk factor for coronary heart disease (CHD) and increasing concentrations of HDL-C are able to reduce CHD occurrence even in the presence of elevated LDL-cholesterol (LDL-C) levels.^8^ Until now, based on proatherogenic evidences of LDL-C, the therapeutic approaches for CD primary and secondary prevention have been mainly finalized to LDL-C lowering and have reached success especially with statins. In fact, a reduction in baseline LDL-C levels (∼120–190 to 100–140 mg/dL) by statin treatment has been associated with a significant reduction (∼25–35%) in CVD events.

Nevertheless, similar to all drugs, statins presents a number of well-known side effects.^8^ Thus, nowadays, there is still a tremendous need in finding novel substances endowed with antioxidant and LDL-C-lowering powers, and a safer toxicological profile than current experimental therapeutics.

In this regard, several studies have long since suggested benefits of apples on lipid levels. In fact, the old adage “an apple a day keeps the doctor away” has encouraged many scientists to look for the “magic” ingredients responsible for its medical properties. Several studies have led to the hypothesis that polyphenolic compounds, such as quercetin, (−)-epicatechin, (+)-catechin, procyanidins, anthocyanins, dihydrochalcones, or phenolic acids, play a key role in the healthful properties of apples.^9^ In fact, procyanidins have been recently associated with a certain effect on cholesterol metabolism by others and by us.^10,11^ Recently, the administering of Granny Smith apple polyphenolic extract (Applephenon^®^) to mildly hypercholesterolemic subjects for 1 month resulted in a lowering of total cholesterol (TC) and LDL-C by 5% and 8%, respectively, and an increase in HDL-C levels by 5%.^12^ Obviously, these effects are rather low and certainly are not enough for an effective primary CD prevention.

However, we previously demonstrated that different apple species differ in the amount of polyphenolic compounds and Annurca apple, a cultivar native to Southern Italy, was the richest.^10^ These data encouraged us to evaluate the influence of daily consumption of Annurca apples on the cholesterol levels of mildly hypercholesterolemic healthy subjects. A monocentric, randomized, parallel group, placebo-controlled 4-month study was conducted. The subjects (*n* = 250) were randomly assigned to five treatment groups (each 1 of 50 subjects, 28 men and 22 women). Four groups were administered one apple/day among the following: Red Delicious, Granny Smith, Fuji, and Golden Delicious. The fifth group was asked to consume two Annurca apples/day, since the weight of this cultivar is on average the half of the commercial ones considered in this study.

Comparing results, Annurca led to the most significant outcomes, allowing a reduction of total and LDL cholesterol levels by 8.3% and 14.5%, respectively, and an increase in HDL-C levels by 15.2% (all *P* < .001).^13^

Prompted by these results, we formulated capsules containing Annurca apple polyphenolic extract microencapsulated with maltodextrins, accounting for 800 mg/day, and registered them as AppleMetS^®^ (AMS). We formulated gastro-resistant capsules due to our previous knowledge about salivary and gastric digestion effects on some polyphenolic components.^14^ Subsequently, a monocentric, double-blind, placebo-controlled 12-week study was conducted on 250 mildly hypercholesterolemic healthy subjects. As a result, AMS, at 1 month from the administration, was able to substantially impact both LDL-C and HDL-C at the same time (about, −37.6% and +49.3%, respectively), decreasing TC by about 24.9%. Last, but not least, accumulating evidences have demonstrated a relevant antioxidant power of apples procyanidins.^15–17^ Bearing in mind this peculiar combination of effects, this is an unprecedented result never obtained with any other nutraceutical or drug and could be of clinical relevance in the CD primary prevention.

## Materials and Methods

### Apple collection

Annurca (*Malus pumila* Miller cv Annurca) apple fruits (each, about 100 g) were collected in Valle di Maddaloni (Caserta, Italy) in October when fruits had just been harvested (green peel). The fruits were reddened, following the typical treatment^18^ for about 30 days, and then analyzed.

### Annurca supplement (AMS) preparation

The Annurca apple supplement used in this study consisted of gastro-resistant capsules containing Annurca apple polyphenolic extract microencapsulated with maltodextrins (400 mg/cps). The extract was encapsulated in gastro-resistant capsules due to our previous knowledge of salivary and gastric digestion of some polyphenolic components.^14^ The product was formulated by the Department of Pharmacy, University of Naples “Federico II” (Naples, Italy), and registered with the name of AppleMetS (AMS). Large-scale production of AMS has been accomplished by MB-Med Company (Turin, Italy). Apples have been extracted with pure ethanol, and the obtained extract solution has been kept at −20°C for 24 h to allow sugar elimination. After centrifugation, the extract has been spray-dried in combination with maltodextrins, obtaining a fine powder, which has been used to formulate gastro-resistant capsules.

### Study population and protocol

Study participants were recruited by the Samnium Medical Cooperative (Benevento, Italy). Patients were enrolled in November 2015. Patients aged 18–83 years were eligible for enrolment if they had the following values of serum cholesterol parameters at baseline: TC, 200–260 mg/dL; HDL-C, 30–45 mg/dL; and LDL-C, 189–206 mg/dL. The subjects were asked to keep their dietary habits unchanged throughout the entire study.

Exclusion criteria were as follows: smoking, obesity (body mass index [BMI] >30 kg/m^2^), diabetes, hepatic disease, renal disease, heart disease, family history of chronic diseases, drug therapy or supplement intake for hypercholesterolemia, drug therapy or supplement intake containing apple polyphenols, heavy physical exercise (>10 h/week), pregnant women, women suspected of being pregnant, women who hoped to become pregnant, breastfeeding, birch pollen allergy, use of vitamin/mineral supplements 2 weeks before entry into the study, and donation of blood less than 3 months before the study.

The subjects received oral and written information concerning the study before they gave their written consent. Protocol, letter of intent of volunteers, and synoptic document about the study were submitted to the Scientific Ethics Committee of AO Rummo Hospital (Benevento, Italy). The study was approved by the committee and carried out in accordance with the Helsinki declaration of 1964 (as revised in 2000). The subjects were asked to make records in an intake-checking table for the intervention study and side effects in daily reports. The study was a monocentric, double-blind placebo-controlled trial conducted at the Samnium Medical Cooperative (Benevento, Italy).

The study duration was 16 weeks: the group underwent 4 weeks of placebo treatment, consisting of administration of identically appearing capsules containing only maltodextrin, followed by 8 weeks of nutraceutical treatment, and 4 weeks of follow-up. Both the examinations and the study treatment were performed in an outpatient setting. Clinic visits and blood sampling were performed after 12 h of fasting at weeks 0, 4, 8, 12, and 16. Subjects were informed not to drink alcohol or perform hard physical activity 48 h before blood sampling. All blood samples were taken in the morning and immediately after measurement of heart rate and blood pressure. Blood samples were collected in 10-mL EDTA-coated tubes (Becton–Dickinson, Plymouth, United Kingdom) and plasma was isolated by centrifugation (20 min, 2200 *g*, 4°C). All samples were stored at −80°C until analysis. Plasma TC, HDL-C, and LDL-C levels were determined using commercially available kits from Diacron International (Grosseto, Italy).

Analyses were performed on a Diacron International Free Carpe Diem, and intraday and interday variations were 1.4% and 1.6% for TC, 1.6% and 2.2% for LDL-C, and 2.0% and 2.3% for HDL-C, respectively. In addition to these four meetings, six standardized telephone interviews were performed every 14 days starting from the first meeting, to verify compliance and increase protocol adherence. In particular, these interviews reminded patients to complete their intake-checking table for the intervention study and record any treatment discontinuation, or adverse events they might have experienced in the meantime (which were also documented regularly on the case report forms during each telephone interview and clinic visit).

All patients underwent a standardized physical examination, assessment of medical history (for up to 5 years before enrolment), laboratory examination, measurement of blood pressure and heart rate, and evaluation of BMI. At each clinic visit, patients had to complete three self-administered questionnaires on quality-of-life aspects, and their diaries were checked for data completeness and quality of documentation to ensure patient comprehension of the diary items.

### Randomization, concealment, and blinding

A total of 250 eligible patients were assigned to the group to receive AMS supplement. AMS supplement and placebo were coded with different colors and given in random order. The code was not broken until all analyses were completed and the results were analyzed statistically. If a patient dropped out before receiving AMS supplement, he or she was replaced by the next eligible patient enrolled at the same center. The concealed allocation was performed by an internet-based randomization schedule, stratified by study site. The random number list was generated by an investigator with no clinical involvement in the trial. Patients, clinicians, core laboratories, and trial staff (data analysts, statisticians) were blind to treatment allocation.

### Study treatments

The group of 250 patients (116 men and 134 women, 30–83 years of age) was instructed to take two capsules of AMS per day (one capsule at lunch and one at dinner). The volunteers enrolled in this study had the following values of serum cholesterol parameters at baseline: TC, 214–254 mg/dL; HDL-C, 30–43 mg/dL; and LDL-C, 150–205 mg/dL. AMS product consisted of gastro-resistant capsules, each containing 400 mg of Annurca apple polyphenolic extract, and was registered as AppleMetS (AMS). Large-scale production of AMS had been accomplished by MB-Med Company (Turin, Italy) with full respect of all the good manufacturing practices. Noteworthy, the dose of AMS supplement (800 mg/day) adopted for the clinical trial was in full accordance with the maximum polyphenolic extract daily intake (1000 mg), through food supplements and novel foods, indicated by the revised form (January 2015) of the Commission Regulation (EC) No. 258/1997, as the safe polyphenolic daily amount compatible with a good health state.

### Study outcomes and data collection

#### Primary and secondary efficacy outcomes

Primary endpoints measured were the variations of TC, HDL-C, and LDL-C, while key secondary outcomes collected during clinic visits were measurements of blood pressure and heart rate, and evaluation of BMI.

All raw patient ratings were evaluated in a blinded manner at the site of the principal investigator. The decision process was performed according to a consensus document (unpublished standard operating procedure) before unblinding to define conclusive primary and secondary efficacy data from a clinical perspective.

#### Safety

Although no specific toxicity studies have been performed herein, mutagenicity tests and acute/subacute toxicity studies have long since demonstrated the safety of polyphenol content of apples both in mice and human beings. Specifically, the Commission Regulation (EC) No. 258/1997 established 1000 mg as maximum polyphenolic extract daily intake in humans. Accordingly, the AMS dose adopted for the trial was of 800 mg/day, an amount reasonably lower than that regarded as safe in humans. Nevertheless, we assessed safety from reports of adverse events as well as laboratory parameters concerning the hepatic and renal function, vital signs (blood pressure, pulse, height, weight, and BMI), and physical or neurological examinations. Safety was assessed over the entire treatment period at weeks 0, 4, 8, 12, and 16, including adverse events occurring in the first 3 weeks after cessation of treatments.

### Statistics

#### Methodology

During the trial, it became apparent that dropouts and incomplete diary documentation created missing data that could not be adequately handled by the intended robust comparison. To deal with the missing data structure, we used a negative binomial, generalized linear mixed effects model that not only yields unbiased parameter estimates when missing observations are missing at random (MAR)^19^ but also provides reasonably stable results even when the assumption of MAR is violated.^20,21^ Patients who did not provide any diary data (leading to zero evaluable days) were excluded from the MAR-based primary efficacy analysis, according to an “all observed data approach” as proposed by White *et al.*^22^ This approach is statistically efficient without using multiple imputation techniques.^23^ Data retrieved after withdrawal of randomized study treatment were also included in the analysis.

Unless otherwise stated, all of the experimental results were expressed as mean ± standard deviation (SD) of at least five replications. Statistical analysis of data was performed by the Student's *t-*test or two-way ANOVA followed by the Tukey-Kramer multiple comparison test to evaluate significant differences between a pair of means. The statistic heterogeneity was assessed by using Cochran's test (*P* < .1). The I^2^ statistic was also calculated, and I^2^ > 50% was considered significant heterogeneity across studies. A random-effects model was used if significant heterogeneity was shown among the trials. Otherwise, results were obtained from a fixed-effects model. Percent change in mean and SD values was excluded when extracting SD values for an outcome. SD values were calculated from standard errors, 95% CIs, *P*-values, or t if they were not available directly.

Previously defined subgroup analyses were performed to examine the possible sources of heterogeneity within these studies and included health status, study design, type of intervention, duration, total polyphenol dose, and Jadad score. Treatment effects were analyzed using PROC MIXED with treatment (placebo, fresh apples, and apple supplement) and period as fixed factors, subject as random factor, and baseline measurements as covariates, and defined as weighted mean difference and 95% CIs calculated for net changes in serum cholesterol and blood pressure values. Data that could not meet the criteria of variance homogeneity (Levenes test) and normal distribution (determined by residual plot examination and Shapiro–Wilks test) even after log transformation were analyzed by a nonparametric test (Friedman). The level of significance (α-value) was 95% in all cases (*P* < .05).

#### Analysis sets

The full analysis set population included all randomized patients and patients who did not fail to satisfy a major entry criterion. We excluded patients who provided neither primary nor secondary efficacy data from efficacy analyses. The per protocol set consisted of all patients who did not substantially deviate from the protocol; they had two characteristics. First, this group included patients for whom no major protocol violation was detected (for example, poor compliance and errors in treatment assignment). Second, they had to have been on treatment for at least 50 days counting from day of first intake (completion of a certain prespecified minimal exposure to the treatment regimen). Hence, patients who prematurely discontinued the study or treatment before day 44 were excluded from the per protocol sample.

#### Determination of sample size

Pilot data from an interventional study by Nagasako-Akazome *et al.*^12^ supported the assumption that the probability to achieve a better result on apple extract than placebo is 0.71. Hence, a sample size of 21 patients in each group would have 77% power to detect the difference between two groups using a two-sided Mann–Whitney U test on a 5% significance level. Assuming a dropout rate of about 37%, 34 patients in each treatment group had to be enrolled.

### Patient involvement

No patients were involved in setting the research question or the outcome measures, nor were they involved in developing plans for participant recruitment or the design and implementation of the study. There are no plans to explicitly involve patients in dissemination. Final results will be sent to all participating sites.

## Results

### Enrolment and subject attrition

Patients were enrolled in November 2015. A total of 352 patients were screened for eligibility, 102 patients (29.0%) did not pass the screening stage and 250 patients were randomized. The most common reason was that patients did not meet the inclusion criteria regarding values of serum cholesterol parameters at baseline (*n* = 41), followed by general refusal to participate for no specific reasons (*n* = 17) and concerns about the protocol, especially fear of placebo (*n* = 7). Some fulfilled exclusion criteria (*n* = 37).

**FIG. 1.**
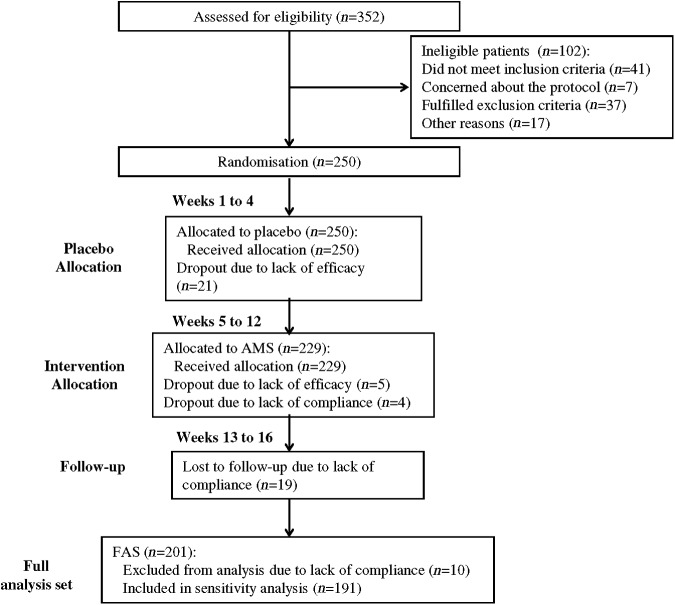
Study flowchart, according to the consolidated standards of reporting trials (CONSORT). The diagram shows enrolment and primary efficacy *endpoints* based on patient diaries, from prescreening to data collection, and the extent of exclusions, loss to follow-up, and completeness of diary documentation available across the entire trial period. AMS, AppleMetS™; FAS, full analysis set.

Overall, 250 patients were assigned to the group assuming the AMS product. Patients underwent a placebo period during the first 4 weeks before the treatment period of 8 weeks. Follow-up period lasted another 4 weeks. Figure 1 shows the flow of participants through the trials together with the completeness of diary information over the entire treatment period.

No patient prematurely terminated study participation before allocation to treatment. Figure 1 follows the CONSORT PRO reporting guideline^24^ and reveals that within the assessment period, the following percentage of patients provided data for the primary endpoint: AMS, 83.4% (191 of 229 patients). A few patients did not submit any diaries, giving no specific reason for this.

### Participants' baseline characteristics

Table 1 shows the demographic and clinical characteristics assessed at the baseline visit of all 250 patients randomized. Overall, about half of the randomized patients were female; the total age range was 18–83 years. The groups were well balanced for demographics and clinical factors.

**Table 1.** Baseline Characteristics of Intention-To-Treat Sample According to Study Treatment

*Placebo*	
*Characteristics*	*AMS (*n* = 250)*
Demographics	
Age (years)	34.7 ± 12.4
Male sex (No. [%])	116 (46%)
White ethnicity (No. [%])	250 (100%)
Clinical parameters (mg/dL)	
TC	234.9 ± 13.1
LDL-C	183.3 ± 11.4
HDL-C	36.2 ± 8.5
Glucose	108.2 ± 8.4
Triglycerides	97.0 ± 9.3

*Treatment*	
Characteristics	AMS (n = 229)
Demographics	
Age (years)	36.1 ± 13.8
Male sex (No [%])	105 (45.8%)
White ethnicity (No [%])	250 (100%)
Clinical parameters (mg/dL)	
TC	233.6 ± 13.7
LDL-C	181.3 ± 12.3
HDL-C	38.0 ± 8.1
Glucose	110.3 ± 9.9
Triglycerides	95.1 ± 8.2

Values are represented as mean ± SD.

AMS, AppleMetS^®^; HDL-C, high-density lipoprotein cholesterol; LDL-C, low-density lipoprotein cholesterol; TC, total cholesterol; SD, standard deviation.

### Primary efficacy outcome measures

No significant variation of plasma TC, LDL-C, and HDL-C levels in subjects belonging to AMS groups at the end of placebo period was registered (Table 1). Analyzing the group supplied with AMS, the results are very exciting as the supplement was able to decrease the mean values of TC and LDL-C by 24.9% (95% CI: −5.36, *P* = .0011) and 37.5% (95% CI: −4.07, *P* = .0021), respectively, while increasing the mean levels of HDL-C by 49.2% (95% CI: −2.14, *P* = .0030). No variation in glucose (CI: −0.64, *P* = .0022) or triglyceride (CI: −0.93, *P* = .0048) levels was registered in patients assuming AMS (Table 2). Interestingly, these results were achieved already after 1 month of the intervention study and confirmed at the end of the second month. Especially, the impact on HDL-C is a result never observed with any other natural or pharmaceutical substance so far. In particular, it is worth to note that the ratio LDL-C/HDL-C in the subject plasma samples decreased from 6.26 to 2.30, meaning a substantial reduction of cardiovascular risk, which is established to maximum levels of 3.0–3.5 (women–men) and 2.5–3.0 (women–men) for the primary and secondary prevention, respectively.^25^

**Table 2.** Effects of Annurca Supplement (AMS) on Plasma Cholesterol, Glucose, and Triglyceride Metabolism

	*AMS*	Δ *(%)*
TC (mg/dL)		
t 0	233.6 ± 13.7	
t 30	176.1 ± 14.2	−24.6
t 60	175.4 ± 13.6	−24.9
LDL-C (mg/dL)		
t 0	181.3 ± 12.3	
t 30	112.7 ± 11.3	−37.8
t 60	113.4 ± 11.6	−37.5
HDL-C (mg/dL)		
t 0	38.0 ± 8.1	
t 30	56.8 ± 7.5	+49.4
t 60	56.8 ± 7.0	+49.2
Glucose (mg/dL)		
t 0	110.3 ± 9.9	
t 30	109.3 ± 10.2	−0.9
t 60	110.8 ± 11.1	+0.4
Triglycerides (mg/dL)		
t 0	95.1 ± 14.2	
t 30	96.2 ± 14.4	+1.1
t 60	95.9 ± 13.3	+0.8

Subjects were administered with two AMS capsules/day for 2 months.

Values are represented as mean ± SD (*n* = 5).

Results were significantly different at a level of *P* = .001.

**FIG. 2.**
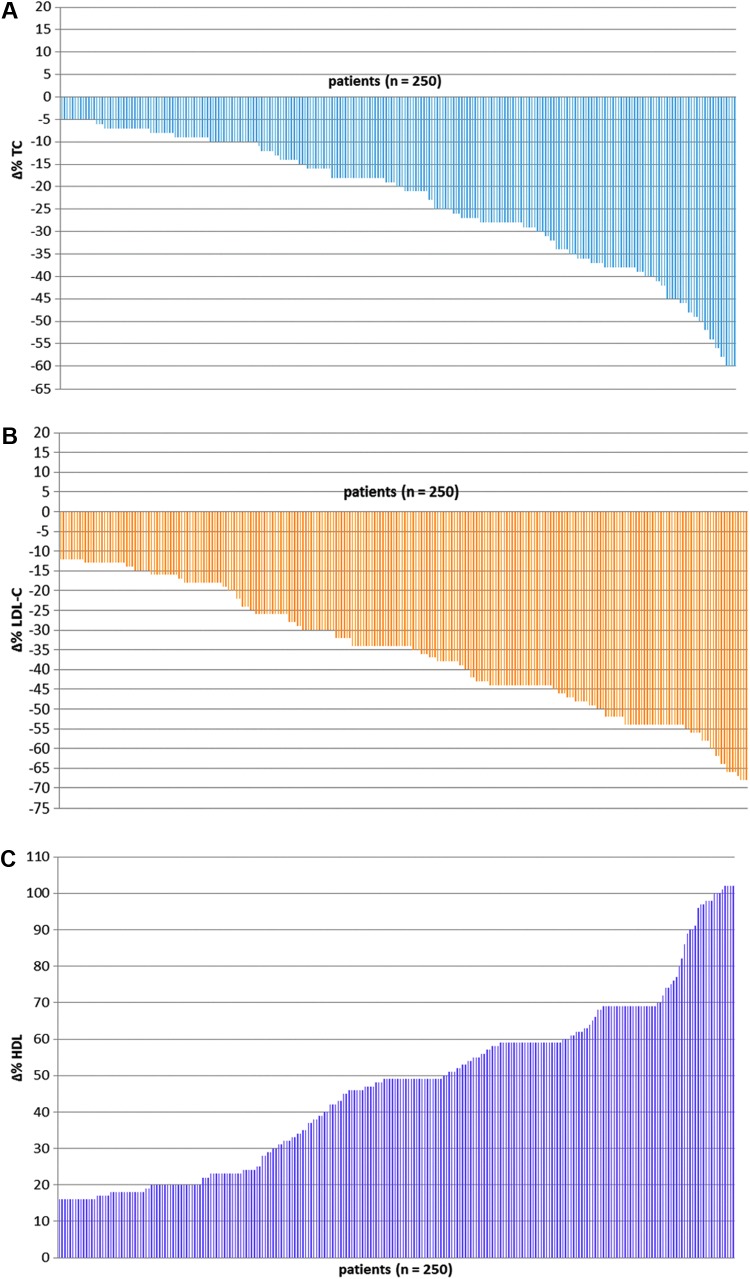
Effects of Annurca supplement (AMS) on **(A)** plasma total cholesterol (TC), **(B)** LDL-C, and **(C)** HDL-C. Subjects were administered with two AMS capsules/day for 2 months. Values are represented as mean ± SD (*n* = 5). Results were significantly different at a level of *P* = .001. HDL-C, high-density lipoprotein cholesterol; LDL-C, low-density lipoprotein cholesterol; SD, standard deviation.

Assuming that a natural remedy, such as a nutraceutical product, may be regarded significantly effective whether capable of favoring a 20% variation of each value of plasmatic TC, LDL-C, and HDL-C, the AMS supplement made possible to 50%, 75.6%, and 85.6%, of the enrolled subjects to reach the respective endpoints (Fig. 2). Moreover, considering that plasmatic values of TC, LDL-C, and HDL-C, regarded compatible with a good health state, may be fixed at 200, 140, and 50 mg/dL, respectively, the clinical trial with AMS supplement made possible to 87.0%, 70.6%, and 68.4% of the enrolled subjects to reach the respective endpoints. Then, we decided to assess if the gender was a variable potentially affecting the AMS efficacy. As a result, analyzing the men and women subgroups, no major differences as regard to the TC (men = 95% CI: −0.85, *P* = .0016; women = 95% CI: −0.63, *P* = .0013), LDL-C (men = 95% CI: −0.74, *P* = .0024; women = 95% CI: −0.84, *P* = .0036), and HDL-C (men = 95% CI: −0.91, *P* = .0012; women = 95% CI: −0.74, *P* = .0022) variations were found (Tables 3–7). Undoubtedly, the most interesting data emerged when the effectiveness analysis of AMS is performed on patients divided in subgroups based either on TC baseline values or on age (Tables 3–7). Beyond the highest expectations, the AMS reveals an increase in efficiency with increase in both patient's age and TC values. Specifically, the most important variations (%) of TC, LDL-C, and HDL-C were revealed within the subgroups characterized by the highest TC value range (Tables 4 and 6) (men 214–229 mg/dL, TC = 95% CI: −2.07, *P* = .0024; HDL-C = 95% CI: −4.67, *P* = .0042; and LDL-C = 95% CI: −2.94, *P* = .0040; men 230–245 mg/dL, TC = 95% CI: −5.77, *P* = .0048; HDL-C = 95% CI: −4.83, *P* = .0018; and LDL-C = 95% CI: −5.22, *P* = .0026; men 246–254 mg/dL, TC = 95% CI: −5.67, *P* = .0038; HDL-C = 95% CI: −3.23, *P* = .0019; and LDL-C = 95% CI: −5.30, *P* = .0034; women 214–229 mg/dL, TC = 95% CI: −5.17, *P* = .0014; HDL-C = 95% CI: −4.60, *P* = .0043; and LDL-C = 95% CI: −5.73, *P* = .001; women 230–245 mg/dL, TC = 95% CI: −4.17, *P* = .0032; HDL-C = 95% CI: −4.23, *P* = .0015; and LDL-C = 95% CI: −5.43, *P* = .0040; and women 246–254 mg/dL, TC = 95% CI: −2.98, *P* = .0028; HDL-C = 95% CI: −4.63, *P* = .0014; and LDL-C = 95% CI: −3.43, *P* = .0048).

**Table 3.** Effects of Annurca Supplement (AMS) on Plasma Cholesterol, Glucose, and Triglyceride Metabolism in Men Subgroup According to Age

	*Men (age: 30–45)*	Δ *(%)*	*Men (age: 46–60)*	Δ *(%)*	*Men (age: 61–83)*	Δ *(%)*
TC (mg/dL)						
t 0	228.6 ± 11.5		233.6 ± 12.8		240.1 ± 13.1	
t 30	174.4 ± 12.5	*−*23.9	175.7 ± 13.8	−24.8	176.2 ± 14.1	−26.9
t 60	174.6 ± 13.0	*−*23.6	175.3 ± 12.3	−25.1	175.7 ± 12.5	−26.8
HDL-C (mg/dL)						
t 0	42.8 ± 3.7		39.1 ± 6.2		34.2 ± 5.3	
t 30	61.4 ± 4.4	+43.2	58.6 ± 5.2	+49.1	54.1 ± 4.8	+58.1
t 60	62.1 ± 3.8	+45.3	59.0 ± 5.1	+50.7	53.3 ± 6.2	+56.2
LDL-C (mg/dL)						
t 0	168.9 ± 13.0		181.1 ± 14.5		192.4 ± 13.8	
t 30	109.1 ± 12.7	−35.4	110.8 ± 12.0	−38.8	112.5 ± 14.4	−41.5
t 60	108.3 ± 12.9	−35.9	111.7 ± 13.8	−38.3	112.0 ± 12.9	−41.8
Glucose (mg/dL)						
t 0	107.2 ± 13.8		112.9 ± 13.6		113.4 ± 11.9	
t 30	106.2 ± 12.5	−0.9	111.2 ± 12.4	−1.2	112.2 ± 13.1	−1.5
t 60	106.7 ± 14.6	−0.5	114.4 ± 10.9	+1.3	114.4 ± 10.3	+0.9
Triglycerides (mg/dL)						
t 0	98.2 ± 10.5		91.1 ± 10.3		99.4 ± 10.3	
t 30	98.5 ± 11.2	+0.3	91.7 ± 12.8	+0.7	100.2 ± 13.0	+0.8
t 60	99.5 ± 11.8	+1.3	91.2 ± 11.6	+0.1	99.6 ± 11.5	+0.2

Subjects were administered with two AMS capsules/day for 2 months.

Values are represented as mean ± SD (*n* = 5).

Results were significantly different at a level of *P* = .001.

**Table 4.** Effects of Annurca Supplement (AMS) on Plasma Cholesterol, Glucose, and Triglyceride Metabolism in Men Subgroup According to Total Cholesterol (TC) at Baseline

	*Men (TC: 214–229)*	Δ *(%)*	*Men (TC: 230–245)*	Δ *(%)*	*Men (TC: 246–254)*	Δ *(%)*
TC (mg/dL)						
t 0	222.1 ± 10.2		233.1 ± 12.3		248.0 ± 12.5	
t 30	169.2 ± 12.5	−23.2	176.3 ± 10.4	−25.3	181.2 ± 14.3	−27.1
t 60	169.7 ± 13.0	−23.0	176.6 ± 11.1	−25.2	181.6 ± 13.1	−27.0
HDL-C (mg/dL)						
t 0	41.1 ± 3.8		38.9 ± 5.3		35.6 ± 4.2	
t 30	59.6 ± 4.2	+45.0	58.7 ± 6.1	+50.3	55.2 ± 4.4	+55.1
t 60	60.0 ± 3.7	+46.1	58.7 ± 4.8	+50.5	55.5 ± 6.1	+55.0
LDL-C (mg/dL)						
t 0	165.3 ± 12.7		180.6 ± 13.1		195.1 ± 12.7	
t 30	104.4 ± 13.2	−36.9	110.6 ± 12.8	−38.9	116.3 ± 13.3	−40.5
t 60	104.8 ± 12.6	−36.3	112.3 ± 12.8	−37.5	117.6 ± 11.7	−39.3
Glucose (mg/dL)						
t 0	109.7 ± 11.1		113.4 ± 11.8		111.2 ± 14.1	
t 30	108.9 ± 12.9	−0.9	112.2 ± 12.5	−1.2	110.5 ± 11.8	−0.8
t 60	109.8 ± 13.6	−0.1	113.7 ± 10.3	+0.3	111.6 ± 12.7	+0.3
Triglycerides (mg/dL)						
t 0	100.2 ± 11.9		90.9 ± 9.3		97.6 ± 11.1	
t 30	101.0 ± 12.4	+0.8	92.6 ± 11.2	+1.7	98.7 ± 11.4	+0.8
t 60	101.5 ± 11.7	+1.3	91.3 ± 10.6	+0.4	98.2 ± 10.2	+0.6

Subjects were administered with two AMS capsules/day for 2 months.

Values are represented as mean ± SD (*n* = 5).

Results were significantly different at a level of *P* = .001.

**Table 5.** Effects of Annurca Supplement (AMS) on Plasma Cholesterol, Glucose, and Triglyceride Metabolism in Women Subgroup According to Age

	*Women (age: 30–45)*	Δ *(%)*	*Women (age: 46–60)*	Δ *(%)*	*Women (age: 61–83)*	Δ *(%)*
TC (mg/dL)						
t 0	226.1 ± 13.6		234.5 ± 11.8		238.4 ± 13.2	
t 30	177.3 ± 10.8	−21.6	177.6 ± 12.5	−24.1	178.5 ± 12.1	−25.0
t 60	176.6 ± 12.0	−21.9	177.3 ± 11.9	−24.4	178.0 ± 13.5	−25.2
HDL-C (mg/dL)						
t 0	40.2 ± 3.2		38.2 ± 5.2		33.8 ± 4.3	
t 30	64.3 ± 5.9	+42.1	56.7 ± 6.4	+48.5	53.1 ± 5.7	+57.2
t 60	56.9 ± 6.1	+41.5	57.5 ± 5.3	+50.6	51.9 ± 3.8	+53.6
LDL-C (mg/dL)						
t 0	170.3 ± 11.5		182.6 ± 13.2		193.5 ± 12.1	
t 30	112.2 ± 13.1	−34.1	113.9 ± 14.1	−37.6	116.1 ± 13.8	−40.0
t 60	111.9 ± 12.4	−34.3	113.5 ± 13.5	−37.8	114.9 ± 11.8	−40.6
Glucose (mg/dL)						
t 0	111.4 ± 12.3		109.2 ± 13.3		105.2 ± 10.2	
t 30	112.2 ± 10.6	+1.0	107.8 ± 14.0	−1.3	104.3 ± 12.3	−0.8
t 60	109.1 ± 10.1	−1.1	110.4 ± 10.0	+1.1	104.8 ± 13.1	−0.4
Triglycerides (mg/dL)						
t 0	88.2 ± 11.1		93.3 ± 12.4		102.4 ± 12.5	
t 30	89.3 ± 12.7	+1.3	91.9 ± 10.7	+0.7	102.7 ± 13.0	+0.3
t 60	88.4 ± 10.1	+0.2	94.3 ± 11.8	+1.1	103.2 ± 11.2	+0.8

Subjects were administered with 2 AMS capsules/day for 2 months.

Values are represented as mean ± SD (*n* = 5).

Results were significantly different at a level of *P* = .001.

**Table 6.** Effects of Annurca Supplement (AMS) on Plasma Cholesterol, Glucose, and Triglyceride Metabolism in Women Subgroup According to Total Cholesterol (TC) at Baseline

	*Women (TC: 214–229)*	Δ *(%)*	*Women (TC: 230–245)*	Δ *(%)*	*Women (TC: 246–254)*	Δ *(%)*
TC (mg/dL)						
t 0	220.0 ± 11.7		232.0 ± 12.5		249.0 ± 12.1	
t 30	172.5 ± 13.5	−21.6	177.1 ± 13.1	−23.7	189.0 ± 13.5	−25.1
t 60	172.0 ± 11.8	−21.8	177.7 ± 10.9	−23.4	188.2 ± 11.5	−25.4
HDL-C (mg/dL)						
t 0	41.2 ± 4.5		37.9 ± 5.5		34.5 ± 4.3	
t 30	59.4 ± 5.2	+44.3	56.7 ± 4.6	+49.7	53.2 ± 5.7	+54.1
t 60	60.0 ± 5.8	+44.8	56.5 ± 5.7	+49.1	53.1 ± 3.8	+54.2
LDL-C (mg/dL)						
t 0	167.4 ± 13.4		182.3 ± 12.6		198.1 ± 12.7	
t 30	110.3 ± 12.5	−34.1	114.7 ± 14.2	−37.1	119.6 ± 11.2	−39.6
t 60	109.0 ± 11.9	−34.9	115.0 ± 12.9	−36.9	120.6 ± 12.7	−39.1
Glucose (mg/dL)						
t 0	114.5 ± 14.1		108.2 ± 12.7		102.7 ± 13.9	
t 30	113.9 ± 12.1	−0.5	107.8 ± 13.9	−0.4	101.3 ± 12.4	−1.4
t 60	115.5 ± 10.4	+1.3	109.2 ± 11.8	+0.9	103.6 ± 14.8	0.9
Triglycerides (mg/dL)						
t 0	95.2 ± 12.5		98.7 ± 13.9		88.7 ± 10.1	
t 30	96.0 ± 11.3	+0.8	98.1 ± 10.5	−0.6	89.3 ± 10.3	+0.7
t 60	95.8 ± 14.7	+0.2	99.4 ± 13.8	+1.3	89.0 ± 10.2	+0.1

Subjects were administered with two AMS capsules/day for 2 months.

Values are represented as mean ± SD (*n* = 5).

Results were significantly different at a level of *P* = .001.

**Table 7.** Effects of Annurca Supplements (AMS) on Plasma Cholesterol, Glucose, and Triglyceride Metabolism in Subgroups Men and Women

	*Men*	Δ *(%)*	*Women*	Δ *(%)*
TC (mg/dL)				
t 0	234.2 ± 12.1		233.0 ± 12.3	
t 30	175.4 ± 14.3	−25.2	177.4 ± 13.1	−23.7
t 60	175.6 ± 12.5	−25.1	176.8 ± 11.9	−24.1
HDL-C (mg/dL)				
t 0	38.6 ± 5.6		37.5 ± 6.4	
t 30	57.9 ± 7.1	+50.0	55.9 ± 3.9	+49.1
t 60	58.0 ± 4.5	+50.4	55.6 ± 7.2	+48.3
LDL-C (mg/dL)				
t 0	180.5 ± 11.3		181.7 ± 12.9	
t 30	110.5 ± 14.0	−38.7	114.5 ± 10.2	−37.0
t 60	112.7 ± 10.8	−37.6	113.9 ± 9.8	−37.4
Glucose (mg/dL)				
t 0	111.4 ± 10.7		108.9 ± 11.3	
t 30	110.4 ± 11.3	−1.0	108.1 ± 10.3	−0.7
t 60	111.7 ± 12.3	−0.3	109.7 ± 11.5	+0.6
Triglycerides (mg/dL)				
t 0	96.3 ± 11.1		94.6 ± 10.4	
t 30	97.4 ± 10.3	+1.2	95.4 ± 12.1	+0.8
t 60	97.1 ± 11.2	+0.8	94.8 ± 11.0	+0.2

Subjects were administered with two AMS capsules/day for 2 months.

Values are represented in mean ± SD (*n* = 5).

Results were significantly different at a level of *P* = .001.

Following a similar trend, the same variations were shown within the subgroups divided into age ranges, highlighting, specifically, major variations for the elderly subjects (Tables 3 and 5) (men 30–45 years old, TC = 95% CI: −3.07, *P* = .0034; HDL-C = 95% CI: −5.57, *P* = .0036; and LDL-C = 95% CI: −3.49, *P* = .0040; men 46–60 years old, TC = 95% CI: −4.38, *P* = .0025; HDL-C = 95% CI: −3.44, *P* = .0039; and LDL-C = 95% CI: −2.44, *P* = .0043; men 61–83 years old, TC = 95% CI: −2.77, *P* = .0028; HDL-C = 95% CI: −4.89, *P* = .0030; and LDL-C = 95% CI: −5.12, *P* = .0026; women 30–45 years old, TC = 95% CI: −4.72, *P* = .0025; HDL-C = 95% CI: −3.42, *P* = .0023; and LDL-C = 95% CI: −6.73, *P* = .0011; women 46–60 years old, TC = 95% CI: −5.61, *P* = .0037; HDL-C = 95% CI: −4.13, *P* = .0015; and LDL-C = 95% CI: −5.28, *P* = .0047; and women 61–83 years old, TC = 95% CI: −3.84, *P* = .0024; HDL-C = 95% CI: −5.63, *P* = .0026; and LDL-C = 95% CI: −3.87, *P* = .0041).

Interestingly, good linear correlations between TC ranges and the entity of variation as regard to TC, HDL-C, and LDL-C were found for the subgroups as follows: men with TC 214–229 mg/dL versus Δ TC, *R* = 0.95; men with TC 230–245 mg/dL versus Δ HDL-C, *R* = 0.98; men with TC 246–254 mg/dL versus Δ LDL-C, *R* = 0.96; women with TC 214–229 mg/dL versus Δ TC, *R* = 0.99; women with TC 230–245 mg/dL versus Δ HDL-C, *R* = 0.95; and women with TC 246–254 mg/dL versus Δ LDL-C, *R* = 0.96. Similarly, good correlations between age range and TC, HDL-C, and LDL-C parameters were found for the subgroups as follows: men 30–45 years old versus Δ TC, *R* = 0.98; men 46–60 years old versus Δ HDL-C, *R* = 0.98; men 61–83 years old versus Δ LDL-C, *R* = 0.96; women 30–45 years old versus Δ TC, *R* = 0.97; women 46–60 years old versus Δ HDL-C, *R* = 0.94; and women 61–83 years old versus Δ LDL-C, *R* = 0.98.

Moreover, taking into account the three main endpoint values (TC, LDL-C, and HDL-C), the entity of variation within age-grouped patients is rather different. Specifically, while the TC variation between the men 30–45 and 61–83 age subgroups is approximately −3%, the change in HDL-C between the same groups is +11% (Tables 3 and 5). Summing up the AMS clinical trial results, we can assert that the AMS formulation (a) strongly impacts the HDL-C plasma values (+49.2%), while decreasing both the LDL-C (−38.1%) and the TC (−24.9%), (b) does not affect at all either the plasma glucose or the triglycerides levels, and (c) is especially effective on elderly people with higher values of baseline TC. These latter data are of outmost importance as elderly people with an unbalanced lipid profile are expected to be the preferential target of any hypolipidemic treatment.

### Safety issues

Although no specific toxicity studies have been performed herein, mutagenicity tests and acute/subacute toxicity studies have long since demonstrated the safety of polyphenol content of apples both in mice and humans being. Specifically, the Commission Regulation (EC) No. 258/1997 established 1000 mg as maximum polyphenolic extract daily intake in humans. Accordingly, the AMS dose adopted for the trial was of 800 mg/day, an amount reasonably lower than that regarded safe in humans. In fact, all the laboratory analyses concerning the hepatic and renal function indicated no alteration of values after two months of AMS treatment (Table 8). Other safety assessments, such as vital signs, blood pressure, or electrocardiographic findings, were all periodically monitored and baseline values did not change substantially during and at the end of the trial.

**Table 8.** Effects of Annurca Supplements (AMS) on Plasma Indicators of Hepatic and Renal Function in Subgroups Men and Women

*Men*												
	*AST (GOT) (U/L)*			*ALT (GPT) (U/L)*			*γ-GTP (U/L)*			*ALP (U/L)*		
	t 0	t 30	t 60	t 0	t 30	t 60	t 0	t 30	t 60	t 0	t 30	t 60
Mean value	21.6	21.5	21.5	27.9	27.5	27.6	37.2	36.2	34.4	222.6	220.4	215.9
*Δ (%)*		−0.5	−0.5		−1.4	−1.1		−2.7	−7.5		−0.98	−3.0

	*LDH (U/L)*			*Albumin (g/dL)*			*Total bilirubin (mg/dL)*			*Creatinine (mg/dL)*		
	t 0	t 30	t 60	t 0	t 30	t 60	t 0	t 30	t 60	t 0	t 30	t 60
Mean value	177.0	176.5	170.2	4.31	4.32	4.16	0.57	0.57	0.54	0.84	0.82	0.80
*Δ (%)*		−0.28	−3.84		+0.23	−3.48		—	−5.26		−2.38	−4.76

*Women*												
	*AST (GOT) (U/L)*			*ALT (GPT) (U/L)*			γ-*GTP (U/L)*			*ALP (U/L)*		
	t 0	t 30	t 60	t 0	t 30	t 60	t 0	t 30	t 60	t 0	t 30	t 60
Mean value	21.8	21.8	21.7	27.3	26.9	27.0	37.4	35.7	34.8	222.8	220.6	214.7
*Δ (%)*		—	−0.46		−1.46	−1.10		−4.54	−6.95		−0.99	−3.63

	*LDH (U/L)*			*Albumin (g/dL)*			*Total bilirubin (mg/dL)*			*Creatinine (mg/dL)*		
	t 0	t 30	t 60	t 0	t 30	t 60	t 0	t 30	t 60	t 0	t 30	t 60
Mean value	179.9	178.8	172.2	4.41	4.32	4.23	0.59	0.57	0.54	0.83	0.80	0.79
*Δ (%)*		−0.28	−3.84		+0.23	−3.48		—	−5.26		−2.38	−4.76

Subjects were administered with two AMS capsules/day for 2 months.

Values are represented as mean ± SD (*n* = 5).

Results were significantly different at a level of *P* = .001.

### Study strength and limitations

The major strengths of the clinical trial herein presented reside in the originality of the study and the evaluation of treatment effects in real-world settings. The positive results, herein reported, can inform physicians about a novel treatment/intervention, which can represent a valuable alternative in the clinical practice. Conversely, the main limitations of our study include the short-term assessment for the treatment of a chronic condition and the choice of exclusively white race.

## Discussion

We previously demonstrated that diverse apple species differ in the amount of polyphenolic compounds and the Annurca apple, a cultivar native to Southern Italy, was the richest.^10^ These data encouraged us to evaluate the influence of daily consumption of Annurca apples on the cholesterol levels of mildly hypercholesterolemic healthy subjects. A monocentric, randomized, parallel group, placebo-controlled 4-month study was conducted. The subjects (*n* = 250) were randomly assigned to five treatment groups (each one of 50 subjects, 28 men and 22 women). Four groups were administered one apple/day among the following: Red Delicious, Granny Smith, Fuji, and Golden Delicious. The fifth group was asked to consume two Annurca apples/day, since the weight of this cultivar is on average the half of the commercial ones considered in this study. Comparing results, Annurca led to the most significant outcomes, allowing a reduction in total and LDL cholesterol levels by 8.3% and 14.5%, respectively, and an increase in HDL-C levels by 15.2% (all *P* < .001).^13^

Prompted by these results, we formulated gastro-resistant capsules containing an Annurca polyphenolic extract and used them for a 2-month clinical trial to evaluate its potential hypocholesterolemic effect in humans (see Materials and Methods, and Results section for details). The novel nutraceutical product, registered as AppleMetS (AMS), has been administered to the enrolled patients in an amount (two capsule/day; 800 mg/day) close to, but lower than that (1000 mg/day) regarded safe in humans by Commission Regulation (EC) No. 258/1997.

Analyzing the lipid profiles of the patients supplied with AMS (800 mg/day) (Table 2), we found that, on average, after 1 month, the TC and LDL-C levels decreased by 24.9% and 37.5%, respectively, while the HDL-C levels increased by 49.2%.

Assuming that a 20% variation in TC, LDL-C, and HDL-C values is a successful endpoint the AMS was effective in 50%, 75.6%, and 85.6% of the enrolled subjects, taking into account the respective three values (Fig. 2). Moreover, considering that plasmatic values of TC, LDL-C, and HDL-C, regarded compatible with a good health state, may be fixed at 200, 140, and 50 mg/dL, respectively, the clinical trial with AMS supplement made it possible for 87.0%, 70.6%, and 68.4% of the enrolled subjects to reach the respective healthy values. Thus, if the solely dietary intervention with apples is not enough for an effective primary CD prevention, according to published literature,^26–29^ the daily administration of a nutraceutical formulation could be of therapeutic significance instead.

Interestingly, our data indicated no major differences between men and women subgroups, as regard to the variations in TC, LDL-C, and HDL-C parameters (Tables 3–7). Undoubtedly, the most interesting data emerged when the effectiveness analysis of AMS was performed on patients divided in subgroups based either on TC baseline values or on age (Tables 3–7). Beyond the highest expectations, the AMS revealed an increase in efficiency with the increase in both patient age and TC values. These latter data are of outmost importance as elderly people with an unbalanced lipid profile are expected to be the preferential target of any hypolipidemic treatment.

Based on our results, we can assert that two capsules of AppleMetS had an LDL-C-lowering effect equivalent to 80 mg of fluvastatin or 40 mg of simvastatin.^30^ Taking into account the dose/effect relationship of atorvastatin, one of the most potent statin available to date, two capsules of AppleMetS provoked a LDL-C-lowering effect analogous to that induced by 10 mg of atorvastatin.^30^ In addition, with respect to statins, which modestly increase HDL-C levels across the range of doses used (5–16%), or to niacin, a GPR109A agonist, which increases HDL-C levels up to 30%, the Annurca nutraceutical (AMS) seemed to be far more effective on the HDL-C raising (+49.2%), and safer. In fact, statin use has been linked to myopathy, type 2 diabetes onset, and, recently, to cancer risk incidence.

The latter side effect clearly emerged in a trial whose aim was to study the effect of simvastatin/ezetimibe (at 40 and 10 mg, respectively) in patients with mild-to-moderate, asymptomatic aortic stenosis.^31^ In the simvastatin–ezetimibe group, incident cancer was diagnosed in 105 patients, compared with 70 patients in the placebo group (*P* = .01). Actually, the safety profile of apple polyphenols has been largely studied on both mice and humans, and, below the 1000 mg/day, no significant hematological, clinical, chemical, histopathological, or urinary effect has been found [Commission Regulation (EC) No. 258/1997)]. In the United States, a Granny Smith apple polyphenolic extract (Applephenon) has been recently approved as generally recognized as safe by the Food and Drug Administration. This product entered the market as nutraceutical product to reduce the serum cholesterol level and prevent obesity. A randomized, human, placebo-controlled intervention study with Applephenon at different dosages showed that the maximum dose (1500 mg/day) resulted in a slight lowering of plasma TC (5%) and LDL-C (8%) levels, while HDL-C increased by 5%.^16^

Dissimilarly, the novel nutraceutical presented in this study, named as AppleMetS, has proven to reduce the TC and the LDL-C levels by 24.9% and 38.1%, respectively, while even more impressive results have been achieved when the HDL-C levels are taken into account (+49.2%). Such high discrepancy clearly depends on the choice of the apple cultivar from which the polyphenolic component has been extracted. Although our study regards the clinical trial devoted to the assessment of AMS hypocholesterolemic efficacy, and thus we did not deepen the exact *in vivo* mechanism of action, we envisage a double antilipidemic effect of procyanidin class. In fact, while dimeric compounds, such as procyanidin B2 would have a statin-like mechanism, as above-mentioned, the polymeric ones, being not readily absorbed, accumulate in the gut, where they act with a β-cyclodextrin-like mechanism, as extensively already demonstrated.^11^

Last, but not least, different from an apple-rich diet, which is known to slightly raise blood glucose and triglycerides (fructose is lipogenic),^32^ the nutraceutical herein presented can exclusively replace the hypocholesterolemic property of apples, without any of the adverse effects of well-known drugs.

In conclusion, herein we presented a monocentric, randomized, double-blind, placebo-controlled 16-week study conducted on 250 mildly hypercholesterolemic healthy subjects, treated first with placebo and then with the AppleMetS formulation. Beyond all expectations, we found that the nutraceutical is fully able to match the modern medicine, while sparing unpleasant side effects of medicines or food itself. In fact, different from an apple-rich diet, the daily assumption of AMS would allow the glucose as well as the triglyceride values not to rise.

What is more, two capsules of AppleMetS have a LDL-C-lowering outcome equivalent to 40 mg of simvastatin or 10 mg of atorvastatin, the latter being one of the most potent statin available to date.^20^ Different from statin-based therapy, the AppleMetS exerts a notable effect on HDL-C levels (+49.2%), while sparing the well-established adverse events such as myopathy, diabetes, or cancer. In this regard, the novel nutraceutical product herein presented is made up by apple polyphenolic extract, whose safeness has long been and extensively demonstrated.

Our work once more demonstrated the value of a severe dietary intervention for CD prevention. However, as things stand in industrialized countries, a drastic change in the population nutrition habits would be as laudable as it is unrealistic. Herein, we offer a simplified remedy with respect to diet transformation or unsafe drug use, with high efficacy and no detectable side effects. Based on the results of the clinical trials, presented in this study, we can assert that AppleMetS formulation could effectively integrate the current therapeutic arsenal to correct impaired lipid profile in humans. Specifically, AppleMetS^®^ may be considered a complementary and/or alternative safe substance, suitable for the treatment of mildly hypercholesterolemic subjects who do not present occurrence of atheromatous plaques yet.
